# New Thiol-Sensitive Dye Application for Measuring Oxidative Stress in Cell Cultures

**DOI:** 10.1038/s41598-018-38132-y

**Published:** 2019-02-07

**Authors:** Virginia Puente-Muñoz, José M. Paredes, Sandra Resa, José Damaso Vílchez, Michal Zitnan, Delia Miguel, María Dolores Girón, Juan M. Cuerva, Rafael Salto, Luis Crovetto

**Affiliations:** 1 Department of Physical Chemistry, Faculty of Pharmacy, University of Granada, Cartuja Campus, 18071 Granada, Spain; 2 Department of Organic Chemistry, Faculty of Sciences, University of Granada, C. U. Fuentenueva s/n, 18071 Granada, Spain; 3 Department of Biochemistry and Molecular Biology II, Faculty of Pharmacy, University of Granada, Cartuja Campus, 18071 Granada, Spain; 4 Materials Research Centre, Faculty of Chemistry, Brno University of Technology, Purkynova 118, Brno, 61200 Czech Republic

## Abstract

A xanthene derivative, Granada Green dinitrobenzene sulfonate (GGDNBS), has been synthesized to assay cellular oxidative stress based on changes in the concentration of biothiols. The dye is able to react with biological thiols by a thiolysis reaction that promotes a change in fluorescence intensity. To demonstrate the usefulness of GGDNBS for *in vivo* oxidative stress measurements, 661 W photoreceptor-derived cells were exposed to light to induce ROS generation, and changes in GGDNBS fluorescence were measured. In these cells, GGDNBS fluorescence was correlated with the biothiol levels measured by an enzymatic method. Therefore, GGDNBS allows us to monitor changes in the levels of biothiols associated with ROS generation via single-cell bioimaging.

## Introduction

Cell damage promoted by unfavorable external factors such as temperature changes, light exposure or/and extreme pH values usually results in the presence of some kind of cellular stress. Such stresses have been associated with the production of high levels of undesirable reactive oxygen species (ROS)^1,2^. Within this context, a wide range of physiological processes at the molecular level are put forward as protective defense mechanisms against the damaging effects of oxidative stress^3^. Among them, a common response is an increase in the levels of biothiol in cellular media, and therefore, one of the most successful methods to measure oxidative stress is to determine the concentration of biothiols.

New methods to measure biothiols are continuously being developed^4,5^. Among them, fluorescence-based approaches are the most interesting ones considering their advantages derived from their high sensitivity, simplicity and low cost^6^. One strategy reported during the last decade for biothiol detection used dinitrobenzenesulfonyl (DNBS) derivatives, and since the first report in 2005, its use has been extended to various other fluorophores^7–13^. The mechanism of action is through the highly selective aromatic nucleophilic addition of thiols to a highly electron-deficient aromatic ring^7^, which releases the fluorophore and hence increases the intensity of the fluorescence signal. Recently, considerable effort has been made to develop new biothiol probes using this strategy, including detecting biothiols in serum and live cells through ratiometric measurements^14^, simultaneously detecting biothiols and phosphate^15^, and using *ex vivo* methods to detect intraperitoneal tumor nodules^16^ or fluorophores with large Stokes shift^17^. Other successful biothiol intracellular probes using different mechanisms of actions, such as a reversible fluorescent biothiol probe^18^, selective detection of GSH over other biothiols such as cysteine or homocysteine in cells^19^, selenocysteine^20^ or selective detection of thiophenols^21,22^ have also been reported.

In this work, we have synthesized a xanthene derivative fluorescent dye, Granada Green dinitrophenyl sulfonate (GGDNBS), which was carefully designed to optimize its intracellular biothiol detection sensitivity and its fast bioimaging response. As a proof of concept to demonstrate its biomedical applications, we have used this probe to measure ROS resulting from light irradiation on photoreceptor cells.

Degeneration of photoreceptors due to oxidative stress^23–25^ is one of the main causes of loss of vision in diseases such as age-related macular degeneration (AMD) or diabetic retinopathy^25–28^. A well-established model for oxidative-stress-induced photoreceptor death is the exposure to light on the mouse-derived photoreceptor cell line 661 W in culture^29^ since it has been demonstrated that short periods of light exposure induce ROS generation and cell death on this cone cell line.

Although the development of reversible and ratiometric probes have been demonstrated very useful in the determination of intracellular biothiols^18,30,31^ including inside mitochondria^32^ we report here the use of a “turn-on” probe.

As expected, our results indicate a dependence on light-induced oxidative stress and intracellular biothiol levels. The use of this fluorescent dye could be optimal for the development of an automated, high throughput method for the screening of new antioxidant drugs for photoreceptor damage-related diseases or other diseases that are associated with an increase in the intracellular ROS concentration.

## Results and Discussion

**Figure 1:**
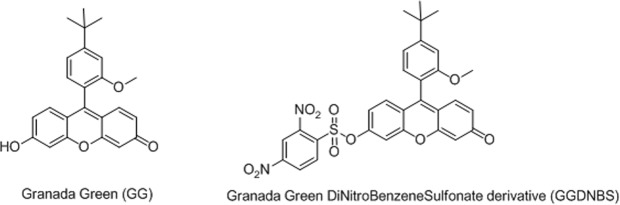
Chemical structures of GG (left) and GGDNBS (right).

We have recently explored the photophysical properties of Granada Green (GG), a xanthenic structure developed in our lab, and its derivatives for the *in vivo* detection of different analytes, including biothiols^33^. Within this context, we now describe a new use of a known group, 2,4-dinitrobenzene sulfonate (DNBS)^7,12,34,35^, in Granada Green (GG) to obtain the derivative GGDNBS (Fig. 1). In contrast to our previous work^15^ where we used a sulfinyl derivative, in this work, we have studied the kinetics and the biological use of the sulfonyl derivative that we selected to monitor the intracellular GSH concentration under stress conditions. The change in the group was motivated by the straightforward synthesis of the sulfonyl derivative, the similar rates of the reaction kinetics (Fig. S1) and the ability to find adequate storage conditions (DMSO as solvent, dark and 4 °C) to avoid the hydrolysis in the stock solutions of the sulfonyl derivative found in our previous work (ethanol as solvent, dark and 4 °C).

Control experiments show that GGDNBS is reasonably stable towards hydrolysis in the presence and absence of N-methyl-D-maleimide (NMM), a well-known biothiol scavenger. Nevertheless, taking into account the high reactivity of GGDNBS, these results do not exclude that other nucleophiles present in the complex cellular media could give a background signal.

**Figure 2:**
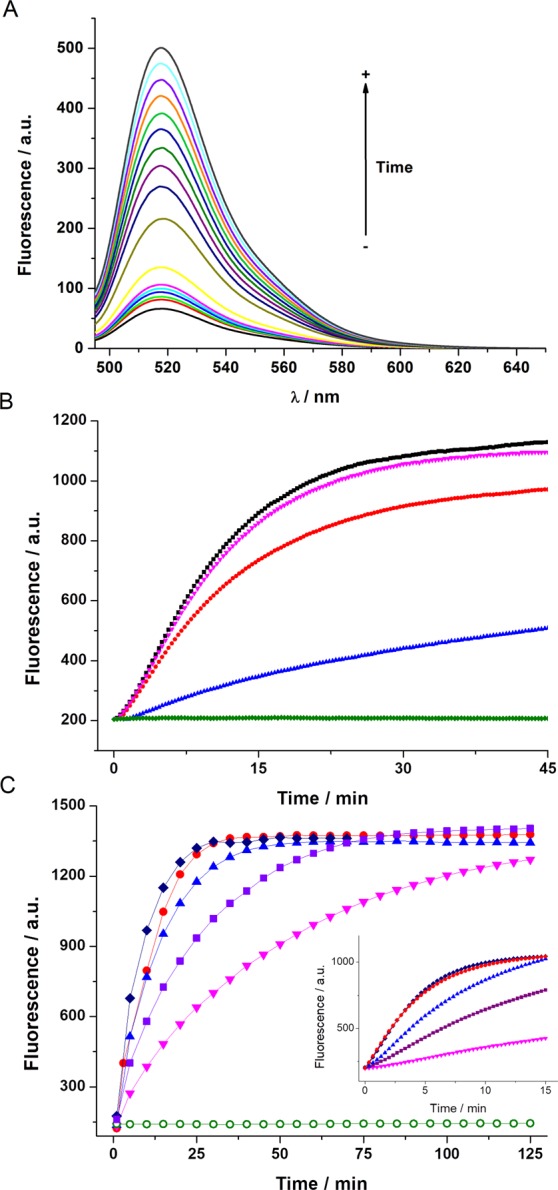
(**A**) Fluorescence spectra of GGDNBS (4 × 10^−6^ M) in Tris buffer (pH 7.35) at 0, 1, 5, 7, 15, 20, 25, 30, 35, 40, 45, 50, 55 and 60 minutes after GSH addition (4 × 10^−4^ M). (**B**) Kinetics of GGDNBS (4 × 10^−6^ M) fluorescence increase after adding GSH (1 mM) (black), GSH (1 mM) + 0.1 mM NMM (pink), GSH (1 mM) + 0.5 mM NMM (red), GSH (1 mM) + 1 mM NMM (blue) and 1 mM NMM (green) in Tris buffer (pH 7.35). The data were obtained using a set excitation/emission of 485/520 nm (**C**) Kinetics of GGDNBS (4 × 10^−6^ M) fluorescence increase after adding GSH at 0 mM (open circles), 0.1 mM (inverted triangles), 0.5 mM (squares), 1 mM (triangles), 3 mM (circles) and 6 mM (diamonds) in Tris buffer (pH 7.35). The data were obtained using a set excitation/emission of 485/520 nm. Inset shows the high resolution time kinetic curves during the first 15 minutes.

As expected, in the presence of glutathione (GSH) (GGDNBS:GSH = 1:100), a notable increase in fluorescence intensity (7.5 times, Fig. 2A) was observed. Moreover, the increase observed was linear during the first 40 minutes (R^2^ = 0.997, Fig. S2). Next, we sought to affirm that the fluorescence increase over time was due to the presence of GSH; for this purpose, we prepared solutions of NMM and biothiols and then added GGDNBS. The use of different GSH:NMM ratios resulted in a dose-response decrease in the fluorescence of the dye. As can be observed in Fig. 2B, a GSH:NMM ratio of 2:1 and even 1:1 blocked the increase in fluorescence due to a lower level of biothiol availability and, consequently, less reaction with the dye. Once we confirmed the specific response of the dye to GSH, we performed a kinetic study in the absence and in the presence of different GSH concentrations ranging from 0 to 6 mM in order to reflect the expected range of intracellular GSH concentration as well as lower GSH concentrations (see Figs S3, S4 and S5). The kinetic data are shown in Fig. 1C, where a dependence of the rate of the reaction on GSH concentration is observed, as well as negligible background hydrolysis in absence of GSH. When the time resolution of the kinetics is high (Figs 2B and S6c), an initial period with low rate was observed and after approximately one/two minutes, the reaction rates increased. To the best of our knowledge, this phenomenon has never been explained before for the thiolysis reaction of this derivative. The S-shape profile could be explained as catalysis promoted by some product of the reaction^36^. To investigate this hypothesis, we added a 4 × 10^−6^ M solution of both reactives (GSH and GGDNBS) and let the reaction for 48 hours to be sure it has finished. Afterwards, we compare the initial rate of two different solutions, one containing both reactives, and the other adding the products of the reaction in a 10-fold lower concentration (4 × 10^−7^ M). Results are shown in Fig. S6F, where no significant change in the rate was observed. Therefore, the observed “lower rate” in the first minutes of the reaction might result from a concurrent mechanism of fluorescence formation that slows down after some seconds and thus only adds in the beginning.

Next, using the rates after the complex initial reaction, we determined the order of the reaction with respect to GSH through the calculation of the initial rates at different GSH concentrations (see SI and Fig. S6A). The orders of the reaction for each reactant were 0.7 ± 0.12 and 0.45 ± 0.05 (with respect GSH and GGDNBS, respectively). Our results show a complex reaction mechanism under our experimental conditions and these novelty results will be deeply studied in future approaches. In addition, more interestingly, we used the area under the curve of the kinetics of the increase in fluorescence for the first 30 minutes to quantify GSH concentration in the medium (see Fig. S6B). Therefore, these results in aqueous solution predict excellent behavior for the potential use of GGDNBS as an intracellular probe in a short period of time.

**Figure 3:**
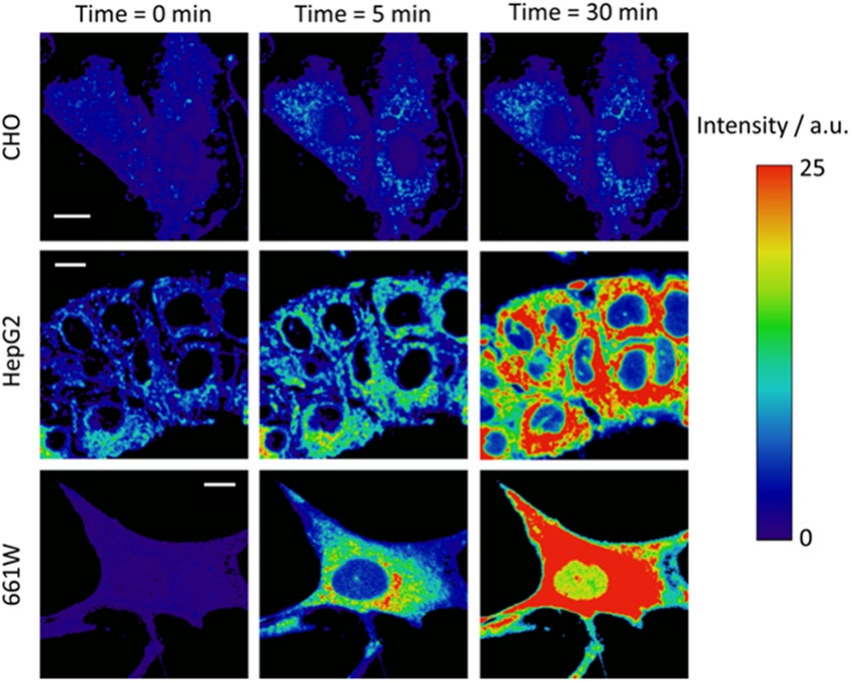
FIM images of GGDNBS (2.43 × 10^−7^ M) in KRP buffer (pH 7.35) in CHO-k1, HepG2 and 661 W cells at different incubation times (0 min, 5 min and 30 min). Scale bars represent 10 μm.

With these results in hand, we examined the GGDNBS response in live cells. The facile cell membrane permeability of GGDNBS allowed the direct use of this compound without the need for any treatment (Fig. 3). This is probably because its lipophilicity allows it to cross the plasma membrane, which is a common behavior for other xanthenic dyes^37,38^. We measured the increase in fluorescence intensity using a control fibroblast cell line, CHO-K1 (Chinese Hamster Ovary Cell line), a human hepatoma cell line, HepG2, and a mouse retinal cone-cell line, 661 W. Figure 3 shows the Fluorescence imaging microscopy (FIM) of the control (CHO-K1) (up), HepG2 (middle) and 661 W (down) cells. As can be observed, at the same measurement times, a significant response is produced in HepG2 and 661 W cells. However, in CHO-K1 cells, where biothiols levels are lower, a negligible increase is observed. To ensure the adequate response of the probe, we measured GSH levels using the enzymatic method (CHO 9.47, 22.1 and 13 nmol/mg in CHO, HepG2 and 661 W, respectively). The results confirm that HepG2 and 661 W cells have a higher content of biothiols in their reservoir pools to protect themselves from the higher production of ROS, which is characteristic of these cell lines^29^. This is in excellent agreement with the data observed in Fig. 3. Therefore, up to this point, the GGDNBS probe shows an adequate ability to discriminate biothiol content in different cell lines.

**Figure 4:**
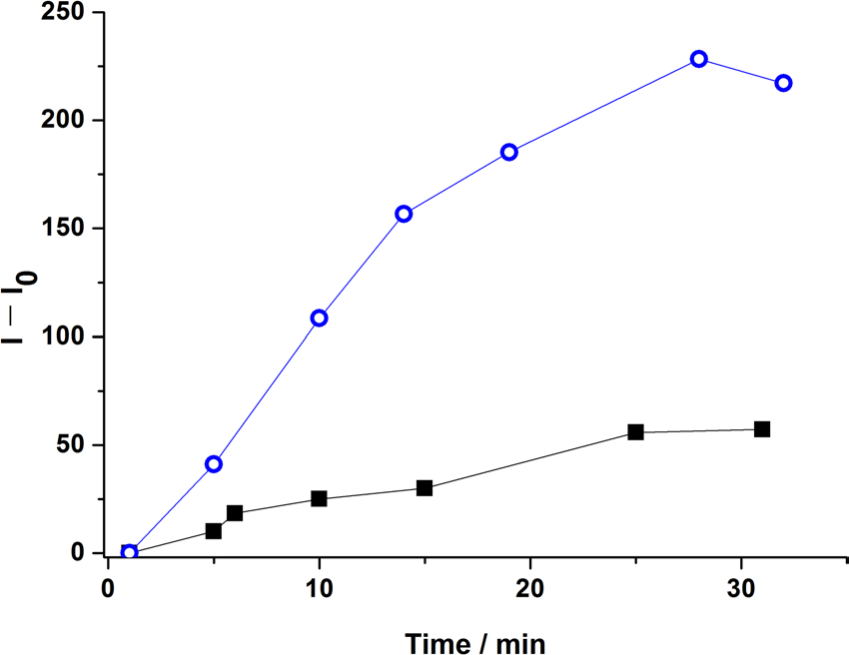
HepG2 cells fluorescence intensity ([GGDNBS] = 2.43 × 10^−7^ M) in the presence (squares) and in the absence (open circles) of NMM (1 mM) in KRP buffer (pH 7.35).

The next step was to confirm the use of GGDNBS as a dynamic biothiol probe in live cells. For that purpose, we studied its response in liver hepatocellular carcinoma (HepG2) cells. As described above, this cell line contains enough biothiols in its reservoir pool to achieve a fluorescence response from GGDNBS. As can be observed in Fig. 4, to confirm the selectivity of the probe towards biothiols in this cell line, we added a thiol−blocking reagent, NMM, to them. The kinetics of the fluorescence increase of the HepG2 cells in the absence (open circles) and in the presence (squares) of NMM is presented in Fig. 4. NMM quenched the increase in fluorescence upon addition of GGDNBS with only a slight increase in the fluorescence signal with time. This intensity rise, which was not observed in the *in vitro* assays, can be explained by intracellular generation of biothiols. Therefore, the selectivity of GGDNBS through the intracellular biothiols can be confirmed.

**Figure 5:**
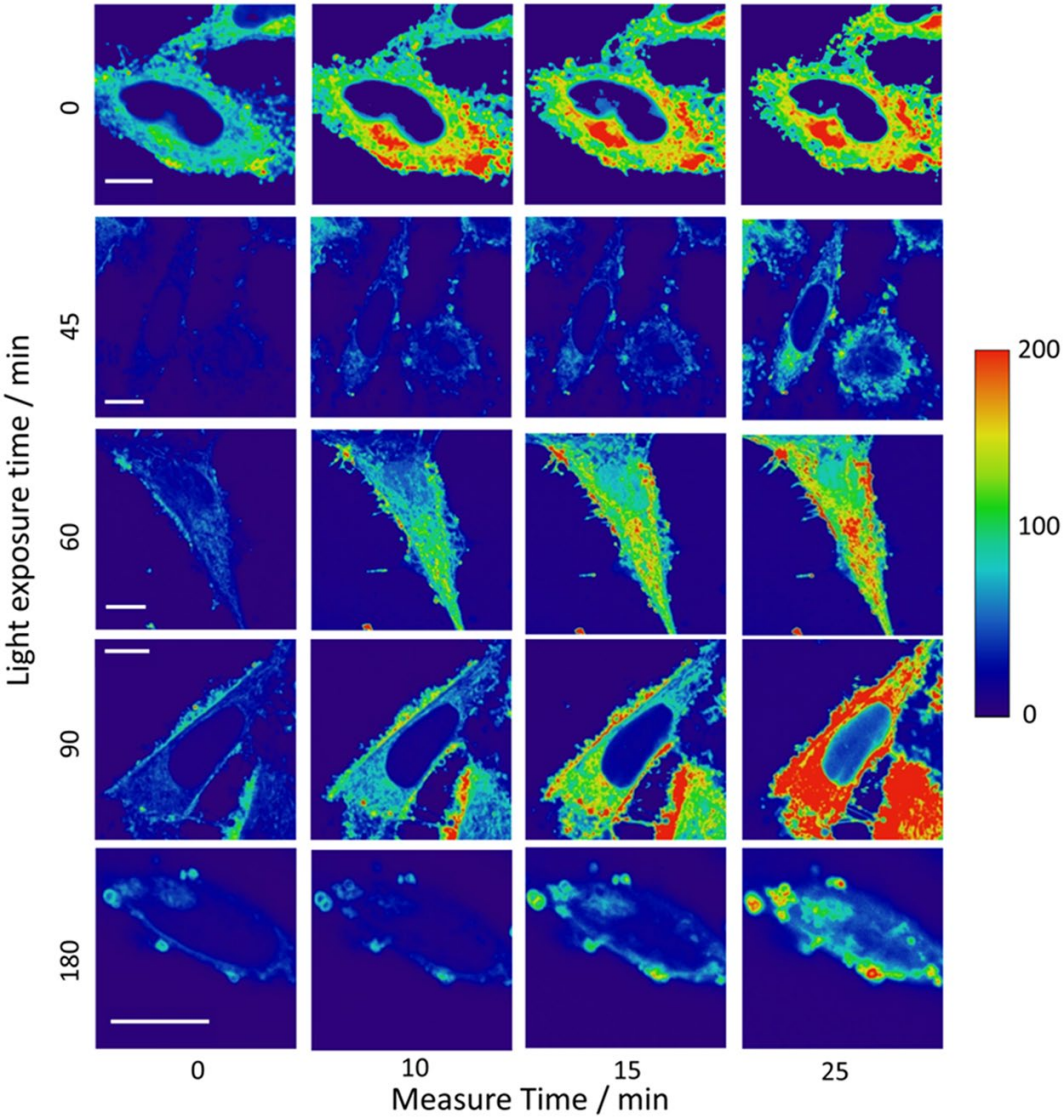
FIM cell images at different light exposure time exposures (0 min, 45 min, 60 min, 90 min and 180 min). The images show fluorescence intensity changes depending on the light exposure time. [GGDNBS] was added at 2.43 × 10^−7^ M in KRP buffer (pH 7.35). Scale bars represent 10 μm.

To demonstrate the ability of GGDNBS to detect rapid changes in biothiol levels caused by oxidative stress, we introduced the dye into 661 W cells. This cell line is used as a model for photoreceptor−like cellular pathologies associated with retinal degeneration caused by light exposure^39^. It has been reported that in this cell line, oxidative stress produces severe damage through photochemical reactions that lead to lipid peroxidation and accumulation of ROS^29^. Therefore, we studied the light−induced cellular stress in 661 W cells. For this purpose, cells were exposed to light during different periods of time (0 min, 15 min, 45 min, 60 min, 100 min, 120 min, 150 min and 180 min), and after light exposure, GGDNBS was added into cells, and the fluorescence intensity was measured by FIM every 5 minutes for 30 minutes. Some of these FIM images are represented in Fig. 5. From each image, the average of the fluorescence intensity values was calculated. As can be observed in Fig. 5, the kinetics of the increase in fluorescence intensity depends on the time elapsed between the light exposure of cell and the FIM measurements. In our analysis, we assume that the intracellular pH does not change. However, an acidification of the cytosol due to oxidative stress promoted by NEM or H_2_O_2_ in cells has been reported^40,41^, and recent measurements give an intracellular pH range between ∼6.9 and ∼7.5 in these conditions^40^. Similar to genetically encoded enzymatic ROS sensors^42^, the probe used in this study is dependent on the pH because the rate of the reaction increases at higher pH and decreases at lower pH (see Fig. S7). Moreover, the released fluorescent dye is in chemical equilibrium between two prototropic species, one of which has negligible fluorescence and the other of which has high fluorescence^33^. However, this latter characteristic can be used to control the changes in intracellular pH according to the maximum intensity performed after the kinetics. Using this approach, we measured the maximum intensity at different light exposure times to estimate changes in the intracellular pH in our experiments. Although this approach does not allow determination of the intracellular pH, it serves as a control to guarantee that we did not detect a drastic acidification in our experiments. Figure S8 shows the maximum intensity value in the kinetics at different light exposure times. The decrease of more than 5% relative to the cell control (at time light zero) indicates a decrease of the intracellular pH and therefore lead to a lower estimation of the real GSH concentration.

**Figure 6:**
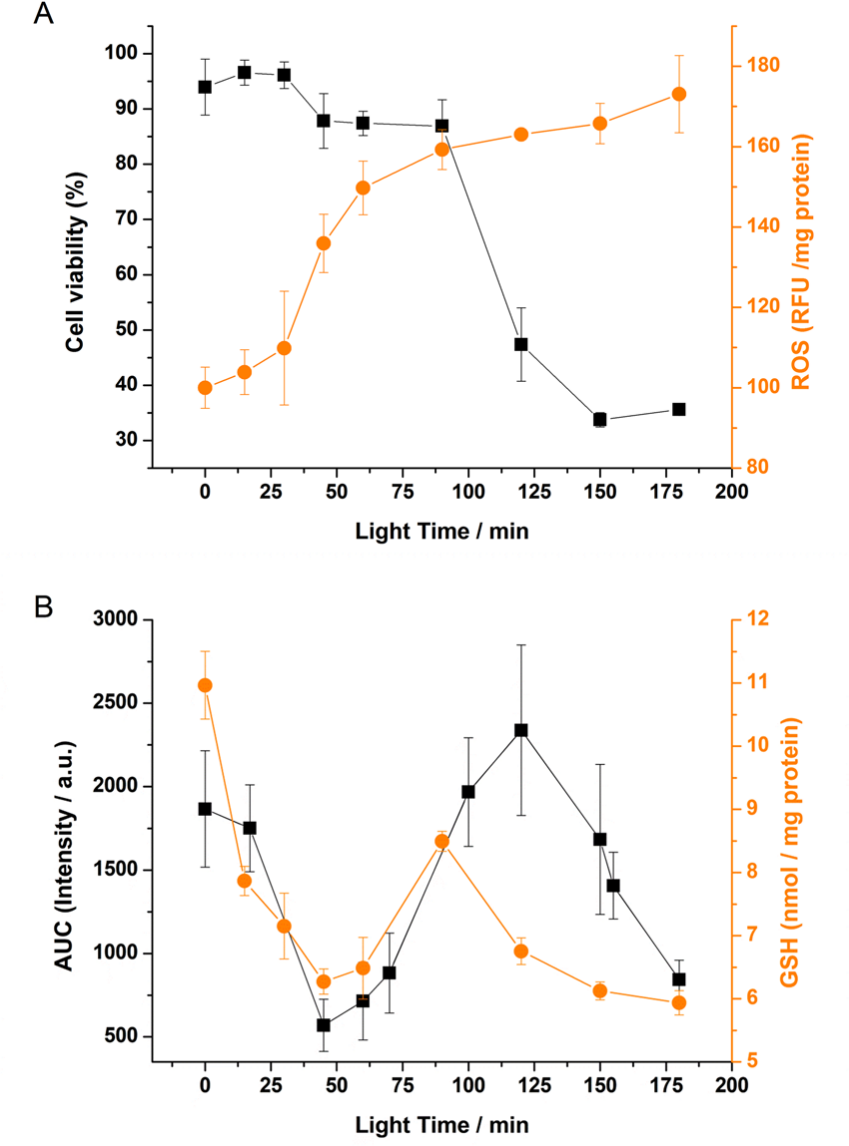
(**A**) Cell viability (black squares) and ROS formation (orange circles) in 661 W cells. 661 W cells were exposed to light for 0–180 min. (**B**) Area under the curve (t = 30 min) from fluorescence intensity data (black square) and GSH enzymatic measurements (orange circles) at different light exposure times. The results are expressed as a means ± SEM. (n = 3 in the AUC and n = 8 in the rest of the data).

To study in depth the dependence of light exposure time on oxidative stress, cell viability and ROS content (assayed using a 2′,7′-dichlorodihydrofluorescein diacetate probe) were measured at selected time points (Fig. 6A). At the same time points, the area under the curve (AUC) of the kinetics obtained from each light exposure time (Fig. S9) using the GGDNBS probe was calculated and compared with the GSH content of the cells, which was measured by using an enzymatic method (Fig. 6B). As expected, Fig. 6A shows a strong dependence of the cell viability and ROS generation on the light exposure time. Although a 2′,7′-dichlorodihydrofluorescein diacetate probe has been previously used to measure ROS levels in 661 W cells^43^ to further confirm the increase in ROS formation under light exposure, an alternative method using the CellROX Green reagent has been assayed. This reagent is weakly fluorescent and upon oxidation and exhibits a strong fluorogenic signal. The oxidized reagent is able to bind to DNA; thus, its signal is located primarily in the nucleus. Cells were exposed to light for 0, 90 and 180 min and incubated with the reagent for 30 min. Figure S10 shows the confocal images of the cells at the selected time points as well as a quantification of the cell fluorescence. An increase in fluorescence corresponding to ROS formation under increasing times of light exposure was observed.

When the AUC for the GGDNBS probe is depicted (Fig. 6B), it can be observed that at short light exposure times (until ∼40 min), a decrease in the AUC recovered from the fluorescence kinetics is detected. This decrease is probably due to an increase in ROS produced by the light-induced cellular oxidative stress and, as a consequence, a decrease in the reservoir pool of biothiols. During this initial period, changes in cell viability were almost not observed. After this initial period and until ∼120 min, a fast increase in the AUC of fluorescence intensity values was observed. Although the time course of biothiol concentration upon light exposure has not been previously described in this cell line, our results suggest an intracellular synthesis of biothiols as a defense mechanism against the high light-induced stress, as previously reported^44^. This period coincides with a slight decrease in cell viability. Finally, there is a final period after ∼120 min that shows a decrease in the AUC. This is in line with a dramatic decrease in the viability of the cells. Here, we hypothesized that after a long exposure time to light and ROS generation, the cellular capability to restore the biothiol pool is exhausted, and then the ROS-induced cellular stress translates into massive cellular death. When the data obtained with the GGDNBS probe is compared with the GSH content of the cells measured by an enzymatic method, a clear resemblance is observed. Remarkably, this method based on GGDNBS provides a more realistic detection and quantification of biothiols than the enzymatic one, as it gives information about single and live cells, whereas the enzymatic method is based on an average value of all the cells (live and dead). This fact is more striking when the cell viability strongly decreases (t = 120 min, Fig. 6A, black squares). Thus, when comparing GSH concentration determined by the enzymatic protocol (Fig. 6B, orange circles) or by GGDNBS method (Fig. 6B, black squares), lower values were obtained with the first approach, as this provides an average among live and death cells, i.e., those able and unable to synthesize biothiols, respectively, thus diminishing their total concentration.

The reaction of dinitrobenzenesulfonyl compounds with thiols has been shown to be catalyzed by the glutathione S-transferase (GST) family of enzymes. In fact, more recently, DNBS-based probes have been proposed to monitor GST activity in cells^45^. For these reasons, we verified the sensitivity of our sensor to GST activity. We added the enzyme to the sensor with GSH and achieved faster kinetics and higher fluorescence intensities when the enzyme was present (see Fig. S11). These data indicate that the probe is sensitive to GST activity as well as the biothiol concentration. Therefore, to elucidate whether the probe was measuring GSH levels or GST activity under our experimental conditions, the expression and activity of GST were measured in 661 W cells after exposure to light for 0–180 min. Light exposure did not change the expression or activity of GST (Fig. S12). This result indicates that the probe is sensing GSH levels rather than GST activity in our experiments.

## Conclusions

We have designed and synthesized a xanthene derivative optimized as a biothiol sensor for fluorescence imaging microscopy. The mechanism of action requires the thiolysis of the sulfonyl group by biothiols, thus producing a fast increase in the fluorescence intensity. Moreover, to the best of our knowledge, for the first time we described a complex mechanism of the thiolysis reaction of a DNBS derivative.

Our experiments confirm that GGDNBS is an excellent tool for detecting rapidly occurring changes in intracellular biothiol levels as a response of cellular oxidative stress. We performed measurements using hepatocellular carcinoma cells (HepG2) and a photoreceptor-derived cell line (661 W), and the results confirm the ability of this dye to detect intracellular biothiol variations as a consequence of cellular stress induced by ROS. Photoreceptor−like cells have a biothiol reservoir to protect themselves from light-induced ROS that is consumed after a period of ∼40 minutes of light exposure time. After this initial period, intracellular synthesis of biothiols is detected as a response of the cells to defend themselves from ROS. Finally, cell death is observed, and consequently, there is a biothiol concentration decrease due to the excess of light−induced oxidative stress.

The simplicity of this approach can be easily extended to develop high-throughput tests for new antioxidant drugs to prevent and/or treat photoreceptor damage-related diseases.

## Experimental Methods

### Instrumentation

Steady-state fluorescence emission spectra were recorded on a JASCO FP−8300 spectrofluorometer equipped with a 150 W xenon lamp for excitation.

Fluorescence imaging microscopy (FIM) was performed on a Pico Quant MicroTime 200 microscope system with an LDH-485 laser excitation source. The light beam was directed onto a dichroic mirror (510dcxr, Chroma) and passed through an oil immersion objective (1.4 NA, 100×) specific to an inverted microscope system (IX-71, Olympus). After passing through the immersion objective, the fluorescence light was filtered by a long-pass filter (500 LP, AHF/Chroma) and directed to a 75-μm confocal aperture. The light was transmitted to a FF01-520/35 bandpass filter (Thorlabs) and focused on single-photon avalanche diodes (SPCM-AQR 14, Perkin Elmer). The data were collected by a TimeHarp 200 TCSPC module (PicoQuant). Raw fluorescence intensity images were acquired by a scanner with a 512 × 512 pixel resolution (time of acquisition ∼4 minutes), exported as matrix data by SymPhoTime software and analyzed by Fiji ([Fiji Is Just] ImageJ)^46^. To obtain the fluorescence intensity of the cells, output matrix data were exported, and a Gaussian smoothing function was applied (s.d. = 2, in pixels). In addition, the background (extracellular pixels) was removed for the calculation.

### Synthesis of GGDNBS

**Figure 7:**
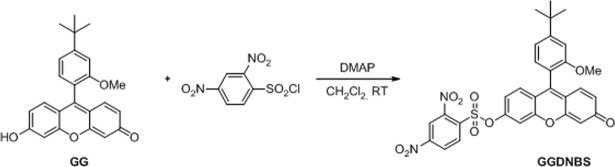
Synthesis of GGDNBS.

The synthesis of Granada Green dinitrobenzene sulfonate (GGDNBS) was carried out starting from the Granada Green probe previously synthesized by our group^15^. We modified and optimized our previous synthetic procedure to exclusively obtain GGDNBS. As shown in Fig. 7, Granada Green (GG, 67 mg, 0.179 mmol) and DMAP (33 mg, 0.269 mmol) were dissolved in 2 mL of dry CH_2_Cl_2_ and then commercially available DNBS-Cl 2,4-dinitrobenzenesulfonyl chloride (DNBS-Cl) (57 mg, 0.215 mmol) was added. The reaction was stirred at room temperature and monitored by TLC. When no starting material was observed, the solvent was then evaporated and residue was purified by flash chromatography (CH_2_Cl_2_: MeOH mixtures). The corresponding product (96 mg, 89%) was obtained as a pure sample, and NMR and HRMS spectra matched with those previously reported^15^.

### Sample preparation

A stock solution of GGDNBS (3.4 × 10^−4^ M) was prepared using DMSO as solvent. From this stock solution, an aqueous (Milli-Q water) solution at a concentration of 1 × 10^−5^ M was prepared to get the desired final concentration of the dye at pH 7.35 in KRP media for cell samples and in Tris (50 mM) for aqueous solution samples. A stock solution of GSH (100 mM) was prepared using diluted HClO_4_ or NaOH to adjust to the desired pH. Commercially available chemicals were used with no further purification. To avoid possible deterioration caused by light and heat exposure, all the solutions were kept cool and in the dark when not in use.

KREBS Ringer Phosphate (KRP) buffer was prepared by mixing the require amounts of different salts. The salts used were the following with the respective concentrations denoted in parentheses: NaCl (118 mM), KCl (5 mM), CaCl_2_ (1.3 mM), MgSO_4_ (1.2 mM), KH_2_PO_4_ (1.2 mM), and HEPES (30 mM). It was checked that the solution had a pH of 7.35.

### CHO-k1, HepG2 and 661 W Cell cultures

Chinese hamster ovary cells (CHO-k1; ATCC no.CCL-61) and human hepatocellular carcinoma HepG2 (ATCC no. HB-8065™) were provided by the Cell Culture Facility of the University of Granada. The mouse retinal cone-cell line 661 W is a transformed cell line derived from mouse retinal tumors and was a gift from Dr. Enrique de la Rosa (CIB, CSIC, Madrid, Spain). All these cells were grown at 37 °C in Dulbecco’s Modified Eagle’s Medium (DMEM) supplemented with 10% (v/v) fetal bovine serum (FBS), 2 mM glutamine plus 100 U/mL penicillin, and 0.1 mg/mL streptomycin. Cells that were submitted to Fluorescence Imaging Microscopy (FIM) were seeded onto coverslips at a density of 2.3 × 10^5^ cells per well into 6-well plates.

### 661 W cells exposure to light and cell measurements

661 W cells were seeded at a density of 2.3 × 10^5^ cells per well into 6-well plates, and incubated for 24 h under a humidified atmosphere of 5% CO_2_ at 37 °C. Then, the cells were exposed to 0.64 mW/cm^2^ of white light from above the 6-well plates for the indicated period of time. Incubations were carried out in a humidified atmosphere of 5% CO_2_ at 37 °C. Control cells were kept in the dark in the same incubator to eliminate any effects due to temperature fluctuations. After light exposition, cells were washed twice with KRP buffer and medium was replaced with KRP buffer. Just before starting the measurement, GGDNBS was added and the measurements were started obtaining an image every five minutes, during 30 minutes.

### Quantitation of light-induced cell death

661 W cells were seeded at 1.5 × 10^4^ cells per well into a p48 plate and 24 h later were exposed to white light for several periods of time as described. Cytotoxicity was assayed by determining the percentage of cell viability (with respect to unexposed cells) using the 3-(4,5-dimethylthiazol-2-yl)-2,5-diphenyl-2H-tetrazolium bromide (MTT)^47^ method, which correlates the cellular metabolic activity with the number of viable cells in culture. The results are reported as % viability based on the untreated control cells at 24 h normalized to 100% viable.

### ROS assay in light-exposed 661 W cells

For the assay of light-induced ROS generation in 661 W cells, cells were seeded at 1.5 × 10^4^ cells per well into a p48 plate and 24 h later were exposed to white light for several periods of time as described. Then, the cells were incubated with 10 µM 2′,7′-dichlorodihydrofluorescein diacetate (DCFH-DA) for 30 minutes, washed twice in PBS, lysed in 50 µl of passive lysis buffer (Promega) and diluted with 150 µl of PBS. The fluorescence generated by the ROS-dependent oxidation of the fluorophore was measured in a JASCO FP−8300 spectrofluorometer at 485 nm excitation and 535 nm emission wavelengths. Protein content was assayed using the Bio-Rad protein assay kit, and the results are shown as relative fluorescence units/mg proteins for each sample.

An alternative method of measuring light-induced ROS generation in 661 W cells was also used. 661 W cells were seeded onto coverslips at a density of 2.3 × 10^5^ cells per well into 6-well plates and 24 h later were exposed to light. Then, cells were incubated with 5 μM of CellROX Green reagent (Molecular Probes, Carlsbad, CA, USA) in the growth medium at 37 °C, washed with PBS three fold and fixed with 3.7% formaldehyde for 15 minutes. Coverslips were mounted on glass slides using Vectashield. Confocal microscopy was performed on a Leica TCS-SP5 II multiphoton confocal microscope. The parameters used to observe CellROX Green were excitation at 488 nm and the emitted fluorescence was detected with a 504–562 nm channel. Data were processed using Leica AF software package.

### GSH determination

For the assay of GSH content in 661 W cells, cells were seeded at 4.5 × 10^5^ cells per p60 plate and 24 h later were treated with white light as described above. At selected time points, cells were trypsinized and washed with ice cold 1X PBS twice and centrifuged at 500 × g. Immediately, the pellets were resuspended with 200 μL ice-cold 5% metaphosphoric acid, mixed, sonicated and centrifuged at 12000 xg for 5 minutes at 4 °C. Supernatants were used for the assay of GSH using a using enzymatic recycling method^48^. Protein pellets were washed with ethanol, resuspended in 0.1% SDS in 400 mM Tris-HCl pH = 8 buffer and assayed for protein content using the Bio-Rad protein assay kit.

### GST activity

661 W cells were exposed to light for 0–180 min. After light exposure, GST activity was measured spectrophotometrically in cell lysates at 340 nm according to Habig *et al*.^49^ using 1-chloro-2,4-dinitrobenzene as a substrate. The protein concentration was measured in cell lysates using the Bio-Rad protein assay kit.

### GST expression

661 W cells were exposed to light for 0–180 min. After light exposure, plates were processed as described in the literature^50^. Proteins were separated by SDS-PAGE and processed for Western blot using an anti-GST antibody (Novus Biologicals, Abingdon, UK) and anti-Gliceraldehyde-3P dehydrogenase (GAPDH) (Santa Cruz Biotechnologies, Inc., Santa Cruz, CA, USA). Immunoreactive bands were visualized by chemiluminescence and quantified with the NIH Image Software.

## Supplementary information

NEW THIOL-SENSITIVE DYE APPLICATION FOR MEASURING OXIDATIVE STRESS IN CELL CULTURES
