# Supplementary material for: New Thiol-Sensitive Dye Application for Measuring Oxidative Stress in Cell Cultures
Source: Sci Rep. 2019 Feb 7;9:1659. doi: 10.1038/s41598-018-38132-y (PMC6367440; doi:10.1038/s41598-018-38132-y)
Supplement: Supplementary file 1 — NEW THIOL-SENSITIVE DYE APPLICATION FOR MEASURING OXIDATIVE STRESS IN CELL CULTURES [file 41598_2018_38132_MOESM1_ESM.docx]

**NEW THIOL-SENSITIVE DYE APPLICATION FOR measuring oxidative stress in cell cultures**

Virginia Puente−Muñoz,^a^ Jose M. Paredes,*^a^ Sandra Resa, ^b^ José Damaso Vílchez ^c^, Michal Zitnan,^d^ Delia Miguel,^a^ María Dolores Girón ^c,^ Juan M. Cuerva ^b^, Rafael Salto^c^ and Luis Crovetto*^a^

a. Department of Physical Chemistry, Faculty of Pharmacy, University of Granada, Cartuja Campus, 18071 Granada, Spain. Correspondence to luiscrovetto@ugr.es, jmparedes@ugr.es

b. Department of Organic Chemistry, Faculty of Sciences, University of Granada, C. U. Fuentenueva s/n, 18071 Granada, Spain

c. Department of Biochemistry and Molecular Biology II, Faculty of Pharmacy, University of Granada, Cartuja Campus, 18071 Granada, Spain.

d. Materials Research Centre, Faculty of Chemistry, Brno University of Technology, Purkynova 118, Brno, 61200, Czech Republic

Index

| Figure S1 | Page 2 |
| --- | --- |
| Figure S2 | Page 2 |
| Figure S3 | Page 3 |
| Figure S4 | Page 3 |
| Figure S5 | Page 4 |
| Kinetic study | Page 4 |
| Figure S6 | Page 6 |
| Figure S7 | Page 8 |
| Figure S8 | Page 8 |
| Figure S9 | Page 9 |
| Figure S10 | Page 10 |
| Figure S11 | Page 10 |
| Figure S12 | Page 11 |

**Figure S1:** Kinetic curves of Granada Green dinitrobenzyl sulfonate (red) (6×10^−6^ M) and Granada Green dinitrophenyl sulfinate (black) (6×10^−6^ M) in aqueous solution at pH 7.35 with GSH (6×10^−6^ M).


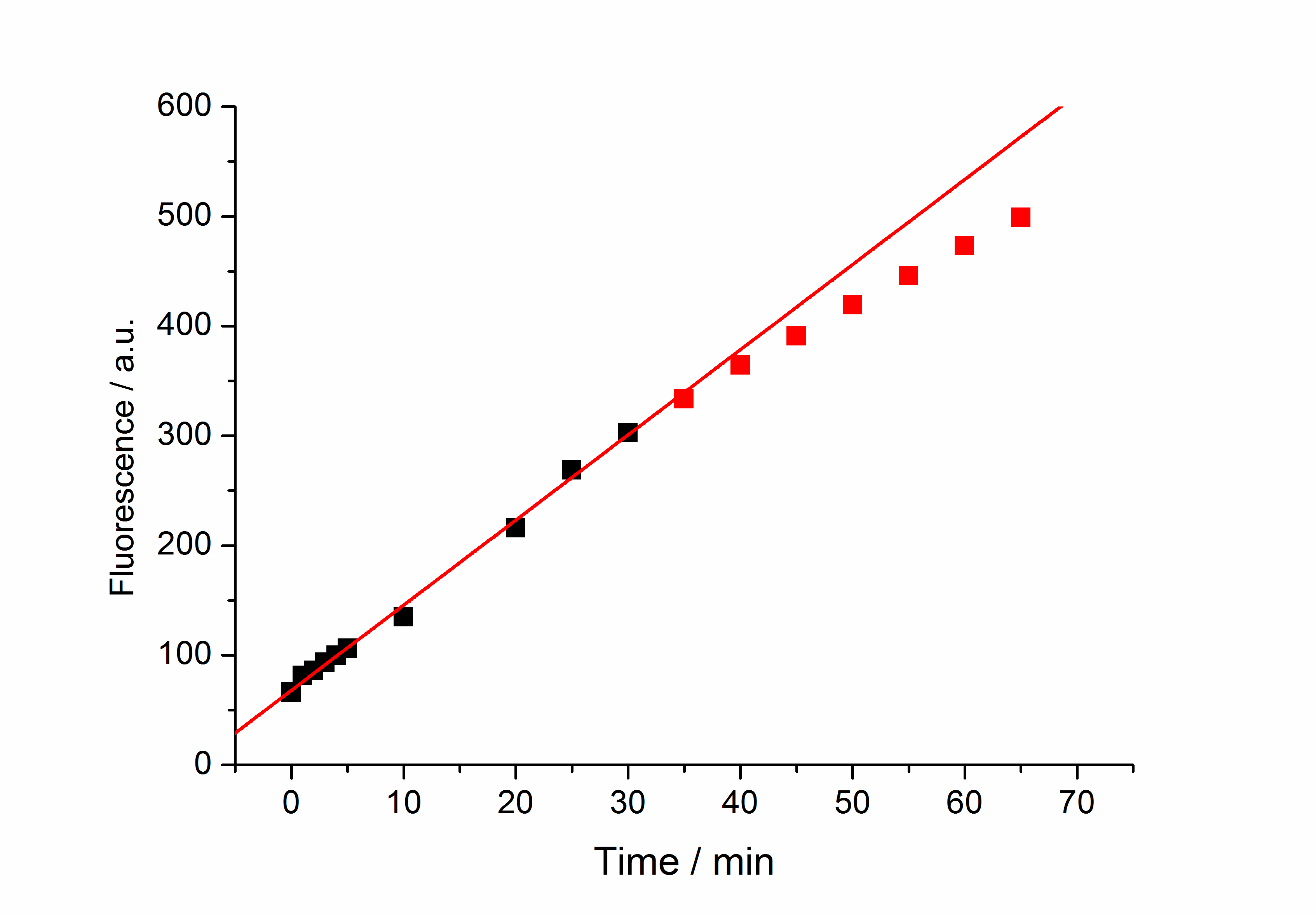


**Figure S2:** Fluorescence increase vs time at 520 nm from 6×10^−6^M of GGDNBS in the presence of GSH (6×10^−4^ M). The linear fit represents the calculation of the initial rate of the reaction.

| A   | B   |
| --- | --- |

**Figure S3:** A) Spectra of fluorescence increase vs time (36 hours) of 6×10^−6^M GGDNBS in the presence of GSH (1:1 ratio). For improved clarity, only ten spectra are shown. B) Maxima of the fluorescence spectra every 5 minutes over 36 hours.

| A   | B   |
| --- | --- |

**Figure S4:** A) Spectra of fluorescence increase vs time (36 hours) of 6×10^−6^M of GGDNBS in the presence of GSH (1:5 ratio). For improved clarity, only ten spectra are shown. B) Maxima of the fluorescence spectra every 5 minutes over 36 hours.

| A   | B   |
| --- | --- |

**Figure S5:** A) Spectra of fluorescence increase vs time (36 hours) from 6×10^−6^M of GGDNBS in the presence of GSH (relation 1:10). For improved clarity, only ten spectra are shown. B) Maxima of the fluorescence spectra every 5 minutes over 36 hours.

Kinetic study

In the kinetic study, we determined the rate law from the experimental data using the differential method. The differential method uses the kinetic equation to calculate the rate through the slopes of the lines representing concentration against time (e.g., Figure 3C)

The proposed reaction equation is:

GGDNBS + GSH → GG + GSDNB + SO_2_

Therefore, the rate (*v*) of the reaction should be given as follows:

$v=k {[GGDNBS]}^{m}{[GSH]}^{n}$ (S1)

where *n* and *m* represent the reaction orders with respect to GSH and to GGDNBS, respectively, and *k* is the apparent kinetic constant. The logarithmic form is then given by:

$$\log v=\log k\left[ GGDNBS \right]^{m}+n\log\left[ GSH \right]$$

Keeping the GGDNBS concentration constant, we can consider an observed rate constant (*k_obs_*) that is given by:

$$k_{obs}=k\left[ GGDNBS \right]^{m}$$

Therefore, we can rewrite the logarithmic form as follows:

$$\log v=\log k_{obs}+n\log\left[ GSH \right]$$

One way to obtain the data is to plot the initial rate of the reaction (*v*_0_) with different initial [GSH]. This technique is known as the initial-rate method. As it has been detected a short initial period with low rate, to calculate the initial rate, we determined the slope of the fluorescence increase from the minute 1.5 until the minute 5. A plot of log *v*_0_ against log [GSH] in this period would be a line with a slope equal to the reaction order with respect to GSH. The observed rate constant can be determined by the intercept. To obtain the data, we used different initial [GSH] (0.1, 0.5, 1, 3 and 6 mM) while keeping the GGDNBS concentration constant.

The plot of the initial rate of the reaction with different initial [GSH] is shown in Figure S6A, and the data are fitted to a linear equation, obtaining a reaction order with respect to GSH of 0.70 ± 0.12 (*n*_GSH_ = 2/3 approx.) and an observed rate constant of (116± 48) M^0.55^ s^−1^

Using an analogous procedure but a constant GSH concentration and increasing the GGDNBS concentration, the logarithmic equations obtained is:

$$\log v=\log{k´}_{obs}+m\log\left[ GGDNBS \right]$$

where ${k´}_{obs}=k\left[ GSH \right]^{n}$

To resolve the system, we measured the initial rates using a fixed GSH concentration of 1 mM and increased [GGDNBS] from 1 to 2, 3, 4 and 6 × 10^-6^ M (Figure S6C). The plot of the initial rate of the reaction at different initial [GGDNBS] is shown in Figure S6D, and the data fit to a linear equation, providing a reaction order with respect to GGDNBS of 0.45 ± 0.05 (*m*_GGDNBS_ = 1/2 approx.) and a *prima* observed rate constant of 73 ± 36 M^0.3^ s^−1^. Fractional orders of a reaction could indicate complex processes in the range of the reactive concentrations used. Once we obtained the orders of the reaction, we estimated an apparent rate constant (*k*) of 4.09 × 10^4^ s^−1^ M^−0.15^. To affirm the validity of our results, we simulated the initial rate of the reaction in the millimolar range of GSH (blue line) and used different micromolar GGDNBS concentrations (black line) and different GSH concentrations. As can be observed in Figure S6E, the concordance with the experimental data is excellent. The analysis of the kinetics have been realized using Origin 8.5, (OriginLab Corp., MA, USA)

| A   | B   |
| --- | --- |
| C  | DD  |
| E   | F |

**Figure S6:** A) Representation of the logarithm of the initial slope of the kinetics from Figure 2C versus the logarithm of the GSH concentration. The data show the average of three different kinetic curves. The error bar represent the standard deviation. B) Area under the curve calculated from the kinetics of Figure 2C. Error bars represent the error estimations for indirect measurements. C) Kinetics of GGDNBS fluorescence increase after adding GSH (0.1 mM) using different GGDNBS concentration: 1×10^−6^ M (squares), 2×10^−6^ M (circles), 3×10^−6^ M (triangles), 4×10^−6^ M (invert triangles), and 6×10^−6^ M (diamonds) in Tris buffer (pH 7.35). The linear plot was used to calculate the rates after the complex initial period, i.e. from the minute 2. In light green color is shown the complex initial region. D) Representation of the logarithm of the initial slope of the kinetics from different concentrations of GDNPS (1 × 10^−6^, 2 × 10^−6^, 3 × 10^−6^, 4 × 10^−6^ and 6 × 10^−6^ M) while maintaining a GSH concentration of 0.1 mM versus the logarithm of the GSH concentration. E) Simulation (lines) of the initial rates using Equation S1 under the same conditions as the experimental data (points) (*n*= 0.70, *m*= 0.45, *k*= 4.09 × 10^4^ s^−1^ M^−0.15^ and [GSH] of 0.1 mM (blue line) and [GGDNBS] of 4 × 10^−6^ M (black line). F) Kinetics of GGDNBS fluorescence increase after adding GSH (0.1 mM), GGDNBS (4 × 10^−6^ M) with (red circles) and without (black squares) the products (4 × 10^−7^ M) of the reaction. Error bars represent the SD.

| A   | B   |
| --- | --- |

**Figure S7:** A) Effect of the pH on the fluorescence kinetics of DNPSGG (4×10^−6^ M) in the presence of 1 mM GSH at pH values of 5.5 (blue triangles), 7.00 (pink invert triangles), 7.35 (black squares) and 7.55 (red circles). B) Maximum intensities (plateau) of the kinetics from Figure S7A.

**Figure S8:** Maximum values of the intensities obtained from the intracellular kinetics at different light exposure times (from 0 to 180 min). Error bars represent the SE. The gray rectangle delimits the 5% variation with respect to the cell control.

| A |
| --- |
| B   |
| C |
| **Figure S9:** Kinetics of average GGDNBS (2.43 × 10^−7^M) fluorescence intensity from FIM images of 661W cells in FRP buffer (pH = 7.35). A) Kinetics after 0 (black squares), 20 (red circles) and 45 (blue triangles) minutes of light exposure time. B) Kinetics after 60 (black squares), 70 (red circles), 90 (blue triangles) and 120 (pink invert triangles) minutes of light exposure time. C) Kinetics after 150 (black squares), 160 (red circles) and 180 (blue triangles) minutes of light exposure time. The arrows indicate the evolution of the kinetics in every stage. Error bars represent the SE. |


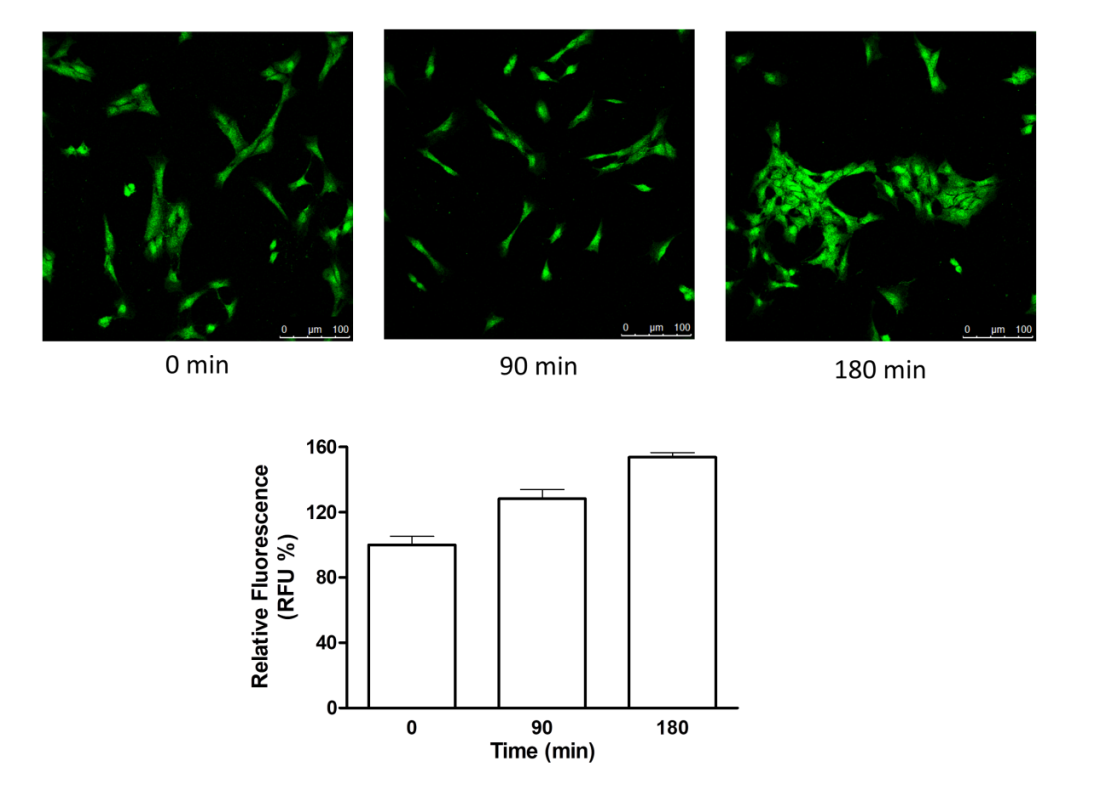


**Figure S10:** ROS measurement in 661W cells. 661W cells were exposed to light for 0-180 min. The cells were stained with 5 μM CellROX Green reagent for 30 min. Then, the cells were mounted for confocal microscopy. Images of the cells as well as quantification of the cell fluorescence is showed. The results are expressed as the means ± SEM (n=12).

**Figure S11:** Fluorescence kinetics of GGDNBS (4×10^-6^ M) in the presence of GSH (4 × 10^-5^ M) in the absence of GST (black square) and after adding GST (0.5 *μ*g/mL) (arrows indicate the addition of GST) in K_2_PO_4_ buffer (pH = 6.5). As can be observed, an increase in the slope and in intensity is achieved after the addition of GST. The data were obtained using a set excitation/emission of 485/520 nm.

| 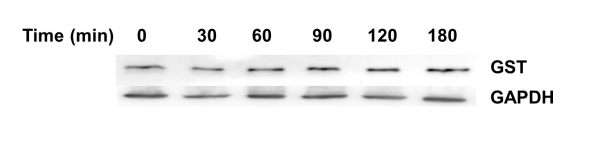A   | B   |
| --- | --- |

**Figure S12:** GST expression and activity in 661W cells exposed to light. 661W cells were exposed to light for 0-180 min. Expression (the expression of GAPDH was used as a loading control) (A) and activity (B) of GST were measured in cell lysates. The results are expressed as the means ± SEM (n=4).
